# Social capital, collective action, and communal grazing lands in Uganda

**DOI:** 10.18352/ijc.761

**Published:** 2017-10-16

**Authors:** Maia Call, Pamela Jagger

**Affiliations:** The National Socio-Environmental Synthesis Center, The University of Maryland, Annapolis, MD, USA; Department of Public Policy, University of North Carolina at Chapel Hill, Chapel Hill, NC, USA

**Keywords:** Collective action, common pool resources, communal grazing land, social capital, Uganda

## Abstract

Recent scholars have found that collective action can be harnessed to sustainably manage common property, contrary to longstanding hypotheses that without effective external regulation community members will exploit communal resources. Researchers have also found that social capital, in addition to biophysical conditions and community attributes, is an important element of successful collective action. However, few studies exploring this topic have specifically examined communal grazing land, which is a critical component of rural livelihoods in many parts of the developing world. To address this gap, we explore the role that collective action plays in maintaining communal grazing lands through bridging, bonding, and linking social capital. In cases where the community does have communal grazing lands, we also explore the role of social capital in determining the condition of the land and the inclusiveness of access. Our analyses draw upon a community-level dataset composed of Uganda RePEAT survey data linked with high resolution gridded socio-environmental data. We observe that strong community bonds are associated with higher odds of successful collective action. However, increased links to external market forces may decrease the odds of successful collective action. These findings provide additional evidence for the complex relationship between social capital, collective action, and common property natural resource management.

## 1. Introduction

There has long been concern that without effective external regulation, community members will abuse communal resources, degrading the environment in order to gain as much of a resource as possible for themselves (Olson 1965). This ‘tragedy of the commons’ (Hardin 1968) scenario has been used repeatedly to hypothesize land degradation and loss of common property resources throughout the developing world, especially in Sub-Saharan Africa. Some research, however, suggests that communities may have the capacity to sustainably manage common property resources through collective action (Pretty 2003). However, studies indicate that communities differ in their ability to harness collective action to sustainably maintain communal resources (Tiffen et al. 1994; Nyangena 2008; Yayneshet and Treydte 2015). In an attempt to explain some of this variation in outcomes, researchers have explored differences in biophysical conditions, attributes of the community and community members, and the rules and norms around communal resources (Ostrom 2011). Though these approaches have been able to explain some differences in communal resource management, they have not been able to fully account for all heterogeneity in communal resource management.

Building on this framework, researchers have additionally examined the role of social capital in collective action around common property. In doing so, these studies have generally found that differing levels of social capital are linked to a community's ability to engage in collective action for the purpose of managing common property (Isham and Kähkönen 1999; Vedeld 2000; Pretty 2003; Nyangena 2008). However, few of these studies have specifically focused on communal grazing land, a vital component of rural livelihoods in many parts of the developing world (Hardin 1968; Mearns 1996; International Livestock Research Institute 1999; Sanginga et al. 2007). Further, those studies that have explored social capital and communal grazing have generally employed descriptive statistics and qualitative methods rather than a robust quantitative approach.

Addressing this gap in the literature, we explore the relationship between social capital and collective action, with the presence of a communal grazing land serving as a proxy for successful grazing land management. For villages with communal grazing land, we also examine the relationship between social capital and inclusiveness of access and condition of grazing land. To investigate these relationships, we analyze a community-level dataset collected in 2003 in Uganda as a part of the Research on Poverty, Environment, and Agricultural Technology (RePEAT) project linked with high resolution gridded socio-environmental data (Yamano et al. 2004). Based on our results, we observe that strong community ties, measured through bonding social capital, are associated with higher odds of successful collective action. Conversely, access to external market forces, indicated through presence of linking social capital, may decrease the odds of successful collective action. These findings have important implications for development programs and policies focused around cattle raising, as they suggest that communities connected to these external programs may have lower odds of having the communal grazing land necessary to benefit fully from an increase in cattle ownership.

## 2. Literature review

### 2.1. Linking collective action, social capital, and common property resource management

Hardin's ‘tragedy of the commons’ posits that in the absence of any formal external governance scheme, users of a common property pasture will intentionally overgraze the land in an attempt to collect the resource before some other user manages to do so (Hardin 1968). This scenario has been highly influential in policy-making for common pool and common property resources at larger scales, often resulting in the establishment of highly centralized or privatized management systems (Ostrom 1990). Though neither are owned by an individual, common property resources differ from common pool resources based on differences in access and withdrawal, management, exclusion, and alienation (Schlager and Ostrom 1992). Common pool resources can be accessed and withdrawn by anyone, are not consistently managed by anyone, and it is difficult to exclude anyone from using them (e.g. the open ocean). Common property resources, however, are typically property held in common and managed collectively by a group of people (Gebremedhin et al. 2004). As such, they are communally managed and may have rules and norms regarding access and withdrawal and the community members may have rights of alienation (e.g. the right to sell the land).

Communal grazing lands, a common property resource, can include extensive tracts of land upon which pastoralists migrate as well as smaller patches of land in a sedentary community (Bekure et al. 1991; Mearns 1996; Pretty and Ward 2001). Regardless of the type and size of communal grazing land, community members have the choice of either competing to appropriate as much of the resource as possible or collaborating through collective action to manage and maintain the resource (Gebremedhin et al. 2004). Successful collective action requires an individual to forgo short-term self-interested behavior in favor of a longer-term group outcome (Rudd 2000). Since the publication of ‘the tragedy of the commons’, researchers have explored the determinants of collective action, discovering many cases in which communities are able to successfully manage common property (Baland and Platteau 1999; Agrawal 2001). The likelihood of successful organization depends on the attributes of the resource(s), the technology for exclusion, the relationship between resources and the user group, the attributes of the user group itself, the ease of noticing if someone is free-riding, the relationship between users and the state, and the presence of social capital (Wade 1987).

Social capital, the focus of our research, can be defined as the shared norms, trust, and horizontal and vertical social networks that enable coordination and cooperation for successful collective action outcomes (Sanginga et al. 2007). The roots of social capital research can be found in Jacobs (1961), Bourdieu (1986), Coleman (1988), and Putnam (1993), who viewed it as representative of the structure of relationships between and among actors. Unlike most forms of capital, social capital is not subtractable and actually increases with further utilization (Putnam 1993). Though more social capital is generally viewed as a net positive for an individual or community, previous research suggests that social capital can sometimes hamper development and that not all aspects of social capital can or should be collapsed into a single unified concept (Woolcock 1998).

To this end, researchers have developed three broad types of social capital: bonding, bridging, and linking. Bonding social capital includes social cohesion within groups arising from relationships between people of similar ethnicity, social status, shared values, or location (Pretty 2003). Bridging social capital can be described as the structural relationships and networks that connect social groups and organizations through collaboration, coordination, social support, or information sharing (Narayan and Pritchett 1999). Linking social capital incorporates the crossing of statuses connecting, for example, those in poverty to those in positions of influence (Pretty 2003). Individually and in concert these types of social capital may contribute to a community's ability to act as a unit, comply with norms, and build connections through local and external organizations and networks. Previous research on the relationship between each form of social capital and collective action provides us with insight on the differing ways in which social capital can be associated with collective action.

### 2.2. Previous studies

Bonding social capital develops within communities through trust and shared moral values. These bonds, such as shared ethnicity, religion, or socioeconomic status, form within a group and help facilitate interaction and collective action (Mearns 1996; Vedeld 2000; Pretty 2003). In their study of the dynamics of social capital and conflict management over natural resource management in the southwestern highlands of Uganda, Sanginga et al. (2007) observe that the effects of social capital are heterogeneous, with different types of social capital contributing unevenly to the prevention and management of conflict. On the other hand, Gebremedhin et al. (2004) find that homogeneity in oxen ownership, a proxy for bonding social capital, does contribute to increased collective action for grazing land management in Ethiopia. Isham and Kähkönen (1999) similarly find that neighborhood trust is an important factor in the successfulness of community-based water projects in Central Java, Indonesia. Further supporting the positive relationship between presence of social capital and successful collective action, Krishna and Uphoff (1999) observe that an index of social capital variables is positively and significantly correlated with the preferred development outcomes in watershed conservation.

Unlike bonding social capital, bridging social capital focuses less on connections within similar groups and more on connections between different groups and individuals (Molinas 1998; Narayan and Pritchett 1999; Nyangena 2008). Isham and Kähkönen (1999) observe that those communities that successfully implemented piped water systems had active groups and associations, and also participated in the design and monitoring of the implemented system. Like Isham and Kähkönen, Gebremedhin et al. (2004) describe the importance of local organizations in promoting collective action for sustainable grazing land maintenance. Communities with a larger number of local organizations contribute more per household for grazing land management and have a higher propensity to use a successful penalty system. Similarly, Gutiérrez et al. (2011) note the importance of connectedness through networks and groups for successful management of fisheries in their meta-analysis of 130 co-managed fisheries in a diverse range of countries.

Linking social capital is similar to bridging social capital. However, linking social capital operates between two individuals or groups that are of different social status, wealth, or influence (Pretty 2003). For example, a connection between a community-level group and an external organization, such as an extension agency or non-governmental organization (NGO), would be a type of linking social capital. Sanginga et al. (2007) find that farmers' groups and women's groups with links to external groups through the local council system have an improved capacity to resolve natural resource management conflicts. In their meta-analysis of biodiversity conservation and forest-based livelihood outcomes, Persha et al. (2011) describe the potential importance of linkages between local institutions and macro-level government institutions for collective action-based sustainable forest management by local communities. Likewise, Pretty and Ward (2001) argue that when governments and NGOs are able to create linkages to the poor, this has the potential to improve the management of natural resources. Molinas (1998) also states that external organizations can support collective action through links with local organizations if the external organizations can provide technical, material, educational, or institutional support. Contradictory evidence comes from Lam (1996) who observes that external organizations may negatively impact collective action if these external organizations increase interest endowment asymmetry among community members. Evans (1996) also points out that external organizations may sometimes crowd out informal networks.

Most of the studies described above have been case studies, employing qualitative methodology or focusing on a limited geographical area. Further, few studies of these explore all three dimensions of social capital in conjunction with one another. Our analysis utilizes large sample survey data and quantitative methods to explore the relationship between all three types of social capital and communal grazing lands in Uganda. Although interest in collective action for sustainable resource management has grown in recent years, there remain a limited number of studies using large-scale broadly representative data for econometric analysis of this topic (Heltberg 2001). The benefit of econometric analysis is that it allows us to identify the relationship between social capital and collective action around communal grazing land while simultaneously controlling for the effect of relevant biophysical and socioeconomic community and household-level variables.

## 3. The Ugandan context

Like many countries in the developing world, Uganda's population is primarily rural, with 87% of Ugandans living outside of urban areas (Uganda Bureau of Statistics 2014). Further, more than 70% of rural Ugandans own livestock (Ministry of Agriculture, Animal Industry and Fisheries et al. 2010). Livestock are generally ranked as the second or third most important livelihood source for rural households, and serve as a number of different forms of capital: physical (labor and consumption), natural (organic fertilizer), financial (security, ‘savings account’), and social (adding to value as a community member) (Ellis 2000; Ministry of Agriculture, Animal Industry and Fisheries et al. 2010). The majority of the rural population subsists through a mixed rainfed crop and livestock system (Ministry of Agriculture, Animal Industry and Fisheries et al. 2010).

Although economic growth in Uganda's agricultural sector is not strong, livestock ownership has been increasing over the past decade. Smallholders with mixed crop and livestock production and pastoralists together own 90% of the cattle in Uganda and almost all smaller livestock. In 2008, the Uganda Bureau of Statistics estimated that there were around 11.4 million cattle in Uganda, more than double the 5.5 million present in 2002 (Uganda Bureau of Statistics 2009). While this increase may be positive from a livelihoods perspective, it has the potential to be devastating from an environmental perspective. While the livestock population is increasing, the total amount of land and resources available for livestock cultivation is finite. Land degradation is of concern throughout Sub-Saharan Africa, though the extent of the degradation is difficult to quantify for methodological and practical reasons (Koning and Smaling 2005). Even without definite quantification of land degradation in the region, it is likely that such rapid livestock population growth will strain the current system (Gebremedhin et al. 2004; Ministry of Agriculture, Animal Industry and Fisheries et al. 2010).

## 4. Methods

### 4.1. Socioenvironmental data

For our analysis, we link household and community survey data with spatial socioenvironmental data. Our survey data come from the Research on Poverty, Environment, and Agricultural Technology (RePEAT) project. The survey was conducted as a joint effort between Uganda's Makerere University, the Foundation for Advanced Studies on International Development, and the National Graduate Institute for Policy Studies (Yamano and Kijima 2010). A project conducted by the International Food Policy Institute (IFPRI) and Makerere University between 1999 and 2001 provided the sampling frame for the RePEAT project (Pender et al. 2004). The IFPRI sample included 107 villages; given the sampling strategy the villages are broadly representative of rural Uganda sampling frame excluded most of northern Uganda, which at the time was affected by conflict. Specifically, 100 villages were selected using a stratified random sample of LC1s (the smallest official administrative unit in Uganda, generally one village) from development domains generated by the researchers though combinations of agro-ecological zones, market access, and population density. The seven additional villages in the sample were selected intentionally from communities in southwestern and eastern Uganda where international researchers are conducting research on land management (Pender et al. 2004).

**Figure 1:**
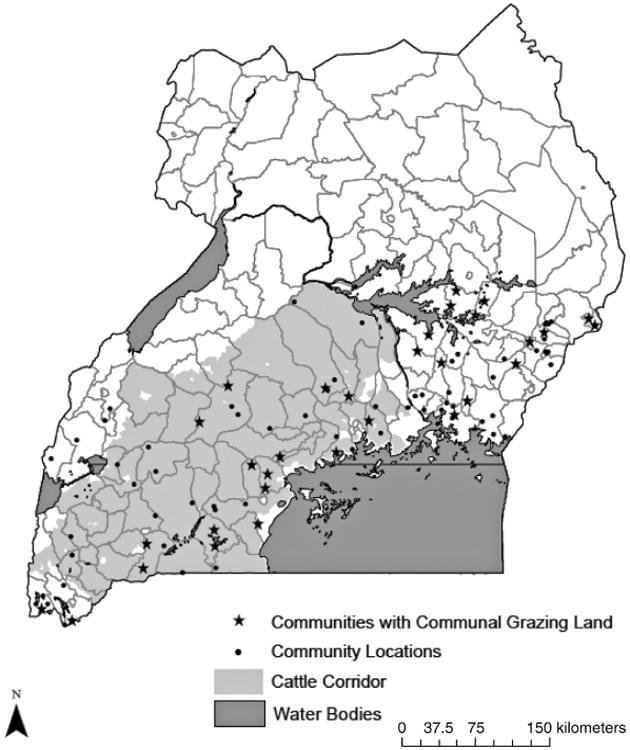
Location of RePEAT study villages, Uganda.

The 2003 RePEAT survey, from which we draw our data, collected data in 29 out of 32 of the original districts and from 94 out of 107 villages (Figure 1). From within each village, 10 households were selected to interview (Yamano et al. 2004). Community surveys were conducted in each village with a group of representative community leaders. Household surveys involved interviewing both household decision makers, and other economically active household members, about many aspects of land management and socioeconomic activity. Respondents reported the presence of communal grazing land in about third of the 94 communities in this study. Communal grazing land has long been a staple of the agro-pastoral livelihood system in Uganda, and most communities have probably had communal grazing at some point. However, only land currently actively managed as communal grazing land was reported as such in the survey.

Table 1 provides a summary of the characteristics of these communal grazing lands at the community and grazing land patch level. Of the 32 communities with communal grazing land, pasture size ranged from 1 acre to 1000 acres, with a median value of 15 acres. Roughly half of the communities with communal grazing lands fell within Uganda's ‘cattle corridor’. In communities with communal grazing land (Figure 1), 1.2% to 100% of community members regularly use a given patch, with the median patch usage of 30% of community members. This measure addresses equitability of access to grazing land, though it is unable to capture the extent to which each household uses the grazing land in a given month. In regard to subjective patch condition, 47.5% of the patches are perceived as in good condition, 9.8% are perceived as in bad condition, and the rest are in average condition. Perceived patch condition was determined through discussion by a group of, on average, ten community members. The vast majority of all the patches are open year-round, not guarded, and do not have use restrictions on what types of animals can be grazed. The rules surrounding appropriate usage of the communal grazing land (such as the types of animals that can graze, length of grazing season, whether the patch is protected and if so, how it is protected) are important in maintenance of communal grazing lands (Gebremedhin et al. 2004). We are unable to examine these rules in our analysis due to lack of variation in the data. The limited presence of formal rule structures around communal grazing suggests that, for Uganda, informal rules and norms governed by social capital are more important than formal rules and sanctions for functional collective action. The primacy of informal rule systems surrounding resource use has important implications for policy makers seeking to formalize existing rule structures (Jagger et al. 2014).

Examining the bonding social capital proxy variables (Table 2) reveals that per household cattle ownership varies greatly across communities, with the median number hovering around three cattle per household. The range of variation is substantial, from a community in which all households own the same number of cattle to a community in which the standard deviation in the number of cattle owned by households is 39.8. The median number of religious groups per community is 3, though some communities are religiously homogenous and some have up to four religious groups. Variation in average wealth within communities is also extensive, while variation in ethnicity is less extreme.

In terms of bridging social capital, mutual aid groups and local organizations offer an opportunity to examine how intracommunity relationships impact social capital. Mutual aid groups are groups wherein the members work to support one another and local organizations can range from farmer's groups to women's organizations. Most communities have at least one mutual aid group and one local organization.

Linking social capital is represented here by NGO representation and the number of micro credit groups in a community. NGOs and microcredit organizations involve local people and typically incorporate outside partners. In developing countries like Uganda, microcredit groups provide poor rural households, which often lack collateral assets, formal employment, and a formal credit history, with access to small loans. These loans can help with community improvement and can provide opportunities for education and entrepreneurship to impoverished households. Similarly, NGOs connect community members with wealthier outside organizations and funders. Communities typically have at least one linkage with a microcredit group, and many communities also have a relationship with at least one NGO.

Additional socioenvironmental data were generated spatially in ArcGIS using a combination of vector and raster data sources. Vector data sources included the boundaries of the ‘cattle corridor’ livelihood zones (Browne and Glaeser 2010) and the locations of the survey communities (Yamano et al. 2004). The forest density (Hansen et al. 2013), population density (Linard et al. 2012), and precipitation (Hijmans et al. 2005) measures were created by extracting average values within a 1 kilometer buffer around the spatial marker for the center of each community. Buffer size was chosen based on exploratory analysis of a related Uganda dataset (Nkonya et al. 2008).

### 4.2. Analytic approach

We employ econometric analysis to examine the relationship between social capital and collective action around communal grazing lands. For our primary model, wherein we identify the factors related to greater odds of the presence of communal grazing land within a community, we use a logistic regression model. In our modeling approach, we first examine the relationship between measures of social capital and odds of grazing land presence in a community without any covariates. Next, we control for property rights, as weak property rights may lead to heavier reliance on communal grazing land. In our third iteration of the model, we leave in measures of land tenure but remove land tenancy for model parsimony. We also include community characteristics associated in the literature with successful collective action. In our final iteration of the model, we also include the spatially derived controls. For examining grazing land condition (good, average, bad) we utilize an ordinal logistic regression approach. To predict community usage, we use a Generalized Linear Model with a logit link and the binomial family, which is appropriate for a dependent variable that is a proportion ranging from 0.1 to 1. For both the grazing land condition and community usage models our sample size is small and so we include only variables in our analysis that have been observed to be significantly related to communal grazing land presence in our primary model or are of particular theoretical importance.

Leveraging data from both the community and household surveys, our indicators of social capital include: (1) *Bonding social capital:* heterogeneity in ethnicity, religion, wealth, and cattle ownership; (2) *Bridging social capital:* number of local organizations and mutual support groups; (3) *Linking social capital:* number of non-governmental organizations (NGOs) involved in the community and number of microfinance groups. Rather than creating indices using these indicators, we include them as individual variables. Our decision is motivated by previous research suggesting that different ways of operationalizing different types of social capital may yield different apparent relationships (Sanginga et al. 2007).

To tease out the apparent relationship between social capital and collective action, we control for variation in property rights regimes, community characteristics, and biophysical features that could impact the likelihood of a community to have communal pasture. Property rights regimes considered here include land tenure (customary, mailo, freehold, leasehold) and land tenancy (owner, occupant, tenant). Customary land tenure is the most common form of land tenure in Uganda. Land holders do not typically have a formal title and may or may not have secure property rights. Mailo land tenure originated with the British colonial government, who rewarded Ugandan colonial agents with large tracts of land. Land held through a freehold can be used, traded, leased, transferred, mortgaged, or donated by the owner, who has a formal title to the land. With leasehold tenure, the land owner has some kind of contract with a tenant giving them rights to use the land for a defined length of time in exchange for payment in cash, labor, or in kind (Kyomugisha 2008). For any of these land tenure types, the tenancy of a given household can be that of owner (full rights over the land), occupant (using the land but not paying a defined rent or other payment), or tenant (renting the land from the owner). The higher presence of less secure (e.g. customary, occupant land tenancy) land rights in a community, the more important it may be for the community to have communal grazing lands. In the case of land loss, communal land can be an important resource for resiliency and recovery (Jagger et al. 2014).

Community attributes include the number of households in the community, the number of cattle per household, amount of land per household, average livelihood diversity, average wealth of the community, and population density. Previous studies have indicated that group size (e.g. number of households) influences success of collective action. For small communities, collective action may be limited because of the expense of collective effort or lack of scarcity of the resource. Large communities have trouble with rule enforcement, competition for the resource, and high transaction costs (Gebremedhin et al. 2004; Jagger et al. 2005). Communities with more cattle may have a greater need of communal pasture. The amount of land per household may also be important, as communities where land is more plentiful may be less in need of a shared pasture. Livelihood diversity may also play a role, with communities with more diverse livelihoods less likely to spend the necessary effort to communally manage a shared grazing land. Wealthier (e.g. asset value, livestock value) communities may not place the same importance on maintaining a communal grazing land, due to access to additional resources, but may do an improved job of maintaining a grazing land if incentivized to do so, for the same reasons. Population density could be linked to communal grazing land by either a lack of land generating need for shared pasture or a lack of land increasing conflict, and therefore decreasing social capital and the likelihood of successfully maintaining a communal grazing land.

Biophysical attributes include driving time to the nearest town, whether a community is located in the ‘cattle corridor’, forest density, and precipitation. Driving time to the nearest town may be related to higher or lower likelihood of collective action. Better market access may increase value of the land and the return from cattle, but increased market access may also decrease community incentives for collective action by providing more exit options (Baland and Platteau 1999). Communal grazing lands are also likely to be more important and possibly more numerous in certain agroecological/livelihood zones in Uganda, where cattle husbandry is a larger part of the typical livelihood strategy (Mwebaze 1999). Agroecological/livelihood zones encompass the ecological conditions (temperature, soil types, topography, and precipitation) as well as the farming systems for a specific region. To control for differences in agroecological zones, we have included measures of forest density, precipitation, and whether or not the community is in the ‘cattle corridor’, a part of southwestern Uganda wherein cattle traditionally play a large role in rural livelihood strategies.

In all regressions, predictors were transformed if they were more normally distributed in an alternative functional form. Data used in this analysis were checked for multicollinearity using the variance inflation factor test. We tested for heteroscedasticity using the Breusch-Pagan test and adjusted for this concern using heteroscedasticity-robust standard errors in our models. Findings from the logistic regression and ordered logistic regression are presented as odds ratios. The odds ratios present the ratio of the odds of the outcome (e.g. grazing land) occurring in a community relative to the odds of it not occurring in the same community. Results from the generalized linear model (proportion of households using grazing land) are presented as relative proportion ratios, wherein for every unit increase in a predictor, the outcome changes by a given percentage.

## 5. Results

Table 3 contains the odds ratios from the presence of grazing land logistic regression (n=94), Table 4 shows the odds ratios from the ordinal logistic regression around patch condition (n=62), and Table 5 reports the relative proportion ratios for the patch usage analysis (n=62). In Table 3, Model 1 contains only the social capital variables, while Model 2 also includes the property rights variables. Model 3 adds in community characteristics while Model 4, the full model, also controls for the biophysical characteristics of each community. Structuring the analysis in this way allows us to observe the impact of theoretically important predictors on the relationship between social capital and collective action for communal grazing land maintenance.

In Table 3, we broadly observe that bonding and linking, but not bridging, social capitals are significantly associated with the presence of communal grazing land. Specifically, the odds of a community having communal grazing land are 73% (p<0.001) lower for every additional religion present in the community. For every unit increase in the variation of cattle ownership, the odds of a community having a grazing land increase by 32% (p<0.1). Conversely, the odds of a community having grazing land increase by 61% (p<0.1) with every unit increase in asset variation. Connections with external microfinance organizations, an indicator of linking social capital, negatively predict the presence of communal grazing land. For every additional microcredit group per 100 households in a community, the odds of a community having grazing land is 32% (p<0.001) lower.

Examining grazing patch condition (Table 4), we find that bonding and bridging social capital are both associated with grazing patch condition. In particular, increased religious diversity decreases the odds that a grazing patch will be in good condition. In regard to bridging social capital, for every additional mutual aid group per 100 households, the odds of a community having good condition grazing land decreases by 66% (p<0.001).

Similar to grazing patch condition, bonding and bridging social capital are both strongly associated with the equitability of grazing patch usage (Table 5). Notably, greater religious diversity and greater variation in cattle ownership are associated with less equitable land usage. Greater variation in assets and livestock values, however, are associated with a higher proportion of households in a community using the communal grazing land. Conversely, a greater number of mutual aid groups in a community is associated with a lower proportion of households using the communal grazing land.

In addition to the social capital measures, we include several property rights, community characteristics, and biophysical controls in our models. Several of these controls proved to be significant, indicating that factors beyond social capital are important for collective action around communal grazing lands in Uganda. Communities with a greater average percentage of land occupied rather than owned or with paying tenants have 5% (p<0.01) greater odds of having communal grazing land. Likewise, communities with greater livelihood diversification have higher odds of having communal grazing land. Location within the cattle corridor and greater amounts of precipitation are both strong predictors of decreased odds of communal grazing land in a community. Population density and driving time to town are not significantly related to presence of grazing land, but do significantly impact grazing land condition and usage. Higher population density is positively related to proportion of community member using grazing land. Increased distance from a market, however, lowers the odds that grazing land will be in good condition and increases the odds that grazing land will be used equitably by the community.

## 6. Discussion

Our findings confirm the importance of bonding and linking social capital as predictors of presence of communal grazing land in communities in rural Uganda. Bonding and bridging, rather than linking, social capitals are associated with grazing patch condition and proportion of community members using a grazing patch. These results support the theoretical and empirical research of previous scholars arguing for the importance of disaggregated measures of social capital (Woolcock 1998).

Examining bonding social capital across all of our models, we find that different proposed measures of bonding social capital have different relationships with our outcomes of interest. Greater religious diversity is associated with lower odds of grazing land presence, poorer condition grazing lands, and lower equitability of grazing land usage. The directionality of this relationship supports claims by previous researchers that cultural heterogeneity has a negative impact on collective action for the maintenance of common property resources (Mearns 1996). Likewise, we observe that greater variation in cattle ownership is associated with lower odds of equitable grazing land usage. On the other hand, we also find that greater variation in household asset values and greater variation in household cattle ownership are associated with greater odds of grazing land in a community and greater variation in household and livestock values are associated with a higher proportion of the community using grazing land. The cross-sectional nature of our analysis makes it difficult to fully understand why these factors, which should theoretically be associated with lower bonding social capital, are associated with positive grazing land outcomes. However, it is very possible that these findings are picking up some additional factor contributing to presence of communal grazing land.

Bridging social capital is not associated with presence of communal grazing land but it is associated with grazing land condition and usage. In particular, communities with more mutual aid groups are significantly less likely to identify their grazing land as in good condition. These communities also tend to have less equitable grazing land usage. These findings are in conflict with previous research suggesting that mutual aid groups may provide community connections that foster successful collective management of resources (Grootaert and Bastelaer 2001). It is possible that communities with greater access to mutual aid groups may be turning their energy to improving the community in ways not picked up through the presence or absence of communal grazing land.

In our analysis, linking social capital is only a significant predictor of grazing land presence in a community. Specifically, communities with a greater number of connections to microcredit groups have lower odds of communal grazing land. This observation is supported by previous research suggesting that external organizations may increase interest endowment asymmetry among community members (Lam 1996) and may crowd out previous established informal networks (Evans 1996).

As described in our results, we found that several socio-environmental community characteristics were significantly related to grazing land presence, maintenance, and usage. Most interesting are those findings related to land tenure and market access. In regard to land tenure, communities wherein land owners allow their land to be occupied, rather than leased, are those where a higher number of the members are related to one another, strengthening bonding social capital. The sharing of land may also be indicative of an overall capacity for collective action. Insecurely occupying property, rather than having a more formal form of land tenure, may also be an incentive to protect common property resources, in case of loss of access to land (Jagger et al. 2014). Lower market access (increased driving time to town) is negatively associated with grazing patch condition but positively associated with equitability of patch usage. Communities further from town may lack access to additional livelihood opportunities not picked up by the livelihood diversity measures, leading those communities to exploit their resources. Conversely, greater distance from the market may lead communities to focus more on customary land management practices and equitable land usage, rather than exploiting these natural resources for the greatest personal gain (Baland and Platteau 1999).

## 7. Conclusions

This study investigates the relationship between social capital and collective action around grazing lands. In contrast with previous studies of this issue, we use a spatially diverse sample and include measures of social as well as biophysical variation in communities. Our findings have important implications for social capital-collective action theory, research methods, and development issues.

Addressing theory, the results highlight the complexity of the relationship between social capital and collective action. Though much research suggests that there is a consistently positive relationship between social capital and capacity for collective action, this study reinforces the importance of considering disaggregated measures of social capital, rather than reducing them to a single metric. Different types of social capital have variable impacts on collective action. Our findings also highlight that property rights regimes and market forces may play a major role in collective action around common property resources.

With respect to methods, we combine spatial analysis with remote sensing and econometrics to develop an analytic approach that we argue is robust and useful for research examining common property natural resource management in the developing world. Key elements include: (1) the use of large-scale stratified random sample of villages that provides us with a fairly high level of external validity; (2) the use of multilevel data that allows us to examine variables derived from the household and community levels; (3) the use of GIS to link communities to environmental and socially important external data, such as precipitation, population density, and livelihood zones; and (4) the use of remotely sensed data to derive the environmental data.

Future research should investigate other measures of social capital as indicators of collective action around communal grazing. Indices of social capital, using questions specially designed for this purpose, would be helpful for exploring this relationship. In addition, it would be useful to be able to econometrically explore the relationship between rules around communal grazing and collective action using a nationally representative, large-sample data source. It was not possible to do so in this analysis due to a lack of variation in those data. These data were also limited by their cross-sectional nature. In order to find potentially causal relationships, it is necessary to use longitudinal data.

Finally, this study has implications for rural livelihoods, development, and poverty in Uganda. Communal grazing lands can be used by everyone, but they are especially important for those who do not have extensive land of their own to graze livestock. If there is indeed a relationship between increasing market connectivity and decreasing condition of common property resources in communities in Uganda, this may lead to disruption of natural resource based rural livelihood strategies. From a development standpoint, providing lower income households with livestock to improve their well-being will only be effective if they have the ability to feed them. Although the number of livestock has increased drastically in recent years, in part because of development efforts, if market expansion and development has a negative impact on the odds of a community having a communal grazing land, these efforts may not be as effective as policy makers and development practitioners may hope. It is important to consider the multifaceted and multidirectional relationships between collective action, social capital, and common property natural resource management if we wish to provide effective development programming to rural communities.

## Figures and Tables

**Table 1:** Descriptive characteristics of communal grazing lands (from community survey).

Characteristic	Community level (n=32)	Patch level (n=62)
Communities in cattle corridor (percent)	59.7%	–
Perceived patch condition (percent)		
Bad		9.80%
Average		42.70%
Good		47.50%
Patch usage by community members (percent)	40%	30%
Area of communal grazing land (hectares)*	24	6
Grazing lands with use restrictions (percent)*		8.06%

Median values represented rather than means to account for left skewed distribution of values.

* Household density per grazing land and use restriction variables were modeled but did not yield significant results, likely due to small sample size.

**Table 2:** Summary statistics.

	Mean	SD	Min	Max	Description
Outcomes					
Communal grazing land (0/1)	0.34	0.48	0	1	l=Presence of grazing land, 0=Absence of grazing land
Grazing patch condition	2.39	0.66	1	3	Grazing patch condition where l=bad, 2=average, 3=good
Grazing patch usage (%)	0.41	0.32	0.012	1	Proportion of households in a community using grazing land
Predictors					
Religious diversity	2.73	0.84	1	4	Number of religions present in a community
Ethnic diversity	2.29	1.43	1	6	Number of ethnicities present in a community
Variation in assets value	2.72	1.74	0	10	Community coefficient of variation of household asset values standardized to 1–10 scale
Variation in livestock value	2.96	2.05	0	10	Community coefficient of variation of household livestock values standardized to 1–10 scale
Variation in cattle ownership	3.82	2.42	0	10	Community coefficient of variation of household cattle ownership standardized to 1–10 scale
Mutual aid groups	1.24	2.11	0	12.12	Number of mutual aid groups per 100 households in a community
Local organizations	0.89	1.58	0	9.68	Number of local organizations per 100 households in a community
Microcredit groups	1.77	3.40	0	23.26	Number of microcredit groups per 100 households in a community
Partnerships with NGOs	0.43	1.48	0	12	Number of partnerships per 100 households in a community
Percentage of land owned	80.22	21.67	6.275304	100	Average percentage of land under owner tenancy in a community
Percentage of land occupied	16.27	21.45	0	93.72	Average percentage of land under occupant tenancy in a community
Percentage of land with tenant	3.51	4.73	0	21.72	Average percentage of land under tenant tenancy in a community
Number of households	162	157	25	1023	Number of households in a community
Average Value of Assets (USD)	88	83	9	566	Average value of assets per household
Average Value of Livestock (USD)	224	300	8	2191	Average value of livestock per household
Average land per household (hectares)	0.72	0.69	0.10	4.05	Average land per household
Livelihood diversity	4.98	1.70	0	10	Index of livelihood diversity in a community in comparison with other communities
Average cattle ownership	5.48	7.21	0	43	Average cattle ownership per household
Population density (people/pixel)	3.10	2.39	0.24	12.82	Population density (1 km buffer)
Driving time to town (hours)	0.87	0.96	0.01	8	Driving time to nearest town
Cattle corridor (0/1)	0.45	0.50	0	1	l=Community is within the ‘cattle corridor’, 0=Community is not within the ‘cattle corridor’
Forest density (pixels)	30.53	11.72	10.21	58.47	Average tree cover across pixels within a community, year 2000 (1 km buffer)
Precipitation (centimeters)	12.26	1.92	8.09	19.60	Climate yearly average of rainfall across pixels within a community (1 km buffer)
Communities=94; Patches=62					

**Table 3:** Logistic regression models predicting presence of grazing land.

	Model 1	Model 2	Model 3	Model 4
Social capital measures				
Bonding				
Religious diversity	0.419*** (0.139)	0.306*** (0.127)	0.437** (0.172)	0.273** (0.140)
Ethnic diversity	0.974 (0.173)	1.043 (0.197)	0.868 (0.171)	0.900 (0.239)
Variation in assets value	1.392** (0.186)	1.453*** (0.202)	1.351 (0.299)	1.613* (0.427)
Variation in livestock value	0.978 (0.192)	1.063 (0.189)	0.847 (0.139)	0.818 (0.164)
Variation in cattle ownership	1.044 (0.144)	1.069 (0.129)	1.194 (0.133)	1.318* (0.221)
Bridging				
Mutual aid groups	0.847 (0.102)	0.842 (0.114)	0.881 (0.140)	0.713 (0.155)
Local organizations	0.824 (0.155)	0.813 (0.153)	0.715 (0.160)	0.792 (0.204)
Linking				
Microcredit groups	0.747*** (0.0803)	0.710*** (0.0805)	0.764*** (0.0721)	0.680*** (0.0766)
Partnerships with NGOs	0.511 (0.211)	0.513 (0.215)	0.448 (0.227)	0.439 (0.247)
Property rights				
Land tenure^1^				
Percentage of land occupied		1.038* (0.0200)	1.015 (0.0125)	1.045** (0.0208)
Percentage of land with tenant		1.014 (0.0560)	1.027 (0.0549)	1.003 (0.0591)
Land tenancy^2^				
Percentage mailo land tenure		0.981 (0.0161)		
Percentage customary land tenure		1.002 (0.0108)		
Community characteristics				
Number of households			0.999 (0.00243)	1.001 (0.00310)
ln(Average value of assets)			1.770 (1.039)	1.311 (1.163)
ln(Average value of livestock)			0.412* (0.197)	0.427 (0.264)
ln(Average land per household)			0.969 (0.297)	0.486 (0.259)
Livelihood diversity			1.486* (0.329)	1.739* (0.521)
Average cattle ownership			1.117* (0.0706)	1.032 (0.0789)
Population density				0.691 (0.208)
Biophysical controls				
ln(Driving time to town)				0.912 (0.347)
Cattle corridor				0.0838* (0.110)
Forest density				0.972 (0.0376)
Precipitation				0.416** (0.169)
Constant	5.173 (5.175)	4.870 (5.865)	27.60 (208.4)	4.244e+08* (4.706e+09)
AIC	119.18	121.45	126.85	117.90
BIC	144.62	157.06	175.17	178.94
Observations	94			

Coefficients are presented as odds ratios, robust standard errors are in parentheses.

1 Tenure base category percentage free/leasehold land tenure.

2 Tenancy base category percentage of land owned.

*** p<0.01,

** p<0.05,

* p<0.1.

**Table 4:** Ordered logistic regression model predicting grazing land condition (bad, average, good).

	Model 1
Social capital measures	
Religious diversity	0.265*** (0.128)
Variation in assets value	1.218 (0.433)
Variation in livestock value	0.844 (0.189)
Variation in cattle ownership	1.130 (0.389)
Mutual aid groups	0.344*** (0.122)
Microcredit groups	1.503 (0.902)
Community characteristics	
Number of households	0.998 (0.00278)
ln(Average value of assets)	0.0700* (0.102)
ln(Average value of livestock)	0.198** (0.139)
Livelihood diversity	1.222 (0.367)
Population density	1.393 (0.735)
Biophysical controls	
Cattle corridor	0.0459*** (0.0547)
Precipitation	0.460 (0.238)
ln(Driving time to town)	0.240*** (0.116)
Observations	62

Coefficients are presented as odds ratios, robust standard errors are in parentheses.

*** p<0.01,

** p<0.05,

* p<0.1.

**Table 5:** Generalized linear model predicting grazing land usage.

	Model 1
Social capital measures	
Religious diversity	0.572*** (0.0838)
Variation in assets value	2.741*** (0.381)
Variation in livestock value	1.438*** (0.139)
Variation in cattle ownership	0.520*** (0.0574)
Mutual aid groups	0.715*** (0.0876)
Microcredit groups	0.775 (0.155)
Community characteristics	
Number of households	0.994*** (0.00110)
ln(Average value of assets)	0.0566*** (0.0253)
ln(Average value of livestock)	4.442*** (0.980)
Livelihood diversity	1.141 (0.158)
Population density	3.010*** (0.554)
Biophysical controls	
Cattle corridor	16.18*** (7.309)
Precipitation	1.723*** (0.191)
ln(Driving time to town)	1.285* (0.171)
Constant	244.6 (1119)
Observations	62

Coefficients are presented as relative proportion ratios, robust standard errors in parentheses.

*** p<0.01,

** p<0.05,

* p<0.1.
